# Depth-Image Segmentation Based on Evolving Principles for 3D Sensing of Structured Indoor Environments

**DOI:** 10.3390/s21134395

**Published:** 2021-06-27

**Authors:** Miloš Antić, Andrej Zdešar, Igor Škrjanc

**Affiliations:** Faculty of Electrical Engineering, University of Ljubljana, Tržaška 25, SI-1000 Ljubljana, Slovenia; andrej.zdesar@fe.uni-lj.si (A.Z.); igor.skrjanc@fe.uni-lj.si (I.Š.)

**Keywords:** depth sensor, line extraction, flat surface extraction, evolving clustering, machine vision, smart sensor

## Abstract

This paper presents an approach of depth image segmentation based on the Evolving Principal Component Clustering (EPCC) method, which exploits data locality in an ordered data stream. The parameters of linear prototypes, which are used to describe different clusters, are estimated in a recursive manner. The main contribution of this work is the extension and application of the EPCC to 3D space for recursive and real-time detection of flat connected surfaces based on linear segments, which are all detected in an evolving way. To obtain optimal results when processing homogeneous surfaces, we introduced two-step filtering for outlier detection within a clustering framework and considered the noise model, which allowed for the compensation of characteristic uncertainties that are introduced into the measurements of depth sensors. The developed algorithm was compared with well-known methods for point cloud segmentation. The proposed approach achieves better segmentation results over longer distances for which the signal-to-noise ratio is low, without prior filtering of the data. On the given database, an average rate higher than 90% was obtained for successfully detected flat surfaces, which indicates high performance when processing huge point clouds in a non-iterative manner.

## 1. Introduction

Modern technologies enable the development of new and more sophisticated sensors, such as stereo cameras, RGB-D sensors, Laser Range Finders (LRF), etc., which are often equipped with powerful processor units to capture and process information-rich data in real time. Due to their increased popularity and ubiquity, they are indispensable in mobile robotics applications [1,2,3,4]. Processing the data obtained from the aforementioned sensors is a computationally demanding task; therefore, it is necessary to ensure the optimal method of processing the acquired data for real-time operations is used, such as vision-based high speed driving [5] or visual inspection in manufacturing processes [6]. With fast and reliable (online) processing of sensor data, which enables high-level representation and perception of the observed environment, we can enable the development of emerging intelligent mobile robots that will be able of autonomous operation, such as fruit picking in an outdoor environment [7] or vacuuming interiors [8]. In this paper, we present methods for planar segmentation of point clouds and attempt to exploit data properties to simplify algorithms while preserving good accuracy.

**Figure 1:**
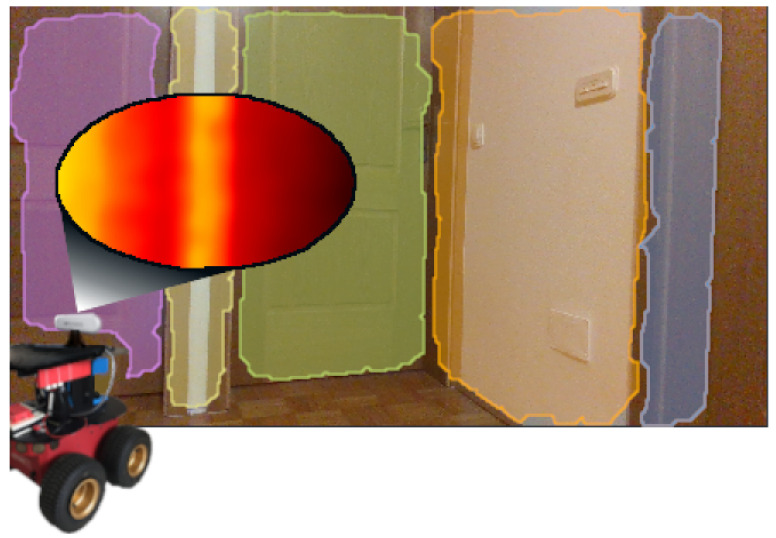
Example of 3D sensing of an indoor environment in the form of flat surfaces, where color represents clusters. Depth image points whose color encodes depth distance are segmented using the EPCC approach.

As shown in Figure 1, planar segmentation enables a more compact representation of the 3D space, where a single point cloud with thousands of points can be described with only a few flat surfaces. Indoor environments are particularly suitable for planar segmentation, as such environments contain many man-made objects that are comprised of flat surfaces. Interior spaces are, therefore, more structured and can be described with prototypes of planes, which are often used as landmarks in autonomous applications, e.g., walking of a quadruped robot on stairs [9]. Various engineering fields utilize 3D perception of the environment in many vision technology applications, such as 3D object detection [10,11] and 3D reconstruction [12,13].

Grilli et al. [14] analyzed the methods and approaches used in point cloud segmentation. They pointed out that 3D segmentation of the point cloud is a difficult task, since the density of point sampling is usually not homogeneous: samples are often disordered and contain significant amounts of noise. In the case of using a depth camera or LRF, we usually obtained organized data, the structure of which allows for a more intuitive, simple, and efficient implementation of the planar segmentation algorithm. However, the problem of uneven point density and the presence of noise remains, especially in stereo camera setups in which the amount of noise increases with distance when measuring depth.

In the field of computer intelligence and machine learning, evolutionary computing is gaining importance as it demonstrates great potential for use in real-time and time-varying systems and environments [15]. Many methods in this area are based on the assumption that a large set of historical data is available for use for model generation in regression or identification. Škrjanc et al. [15] pointed out that this assumption may not hold in real applications, as in these systems the set of previous data may be insufficient, and we usually deal with dynamic environments or systems. They also highlighted the inefficiency of iterative algorithms in processing data that increase with time, as they typically require multiple transitions across the same chunks of data. The described problems are addressed in the field of self-developing modeling [16,17], which solves data stream processing with a self-adaptation approach [18,19], single-pass learning [20,21], and the evolution of model parameters on the fly [22,23].

Certain existing methods exploit the properties of ordered data for hierarchical clustering of planar regions [24] or their extraction based on ordered laser scan profiles [25], but these methods show limitations due to the fixed nature of clustering criteria, which usually lack consideration of nonlinearity of sensory measurements. Methods that take into account the camera noise model [26] have also been proposed to improve segmentation accuracy in short-distance scenes and in wide-open scenes. They proposed robust planar segmentation based on multilateral data smoothing, which, however, does not address the problem of extreme outliers. Data smoothing or filtering processes are typically performed in a batch manner, which is inappropriate for perception algorithms that need to operate in real time. This poses a major problem, especially when processing data from depth cameras. Methods based on the extraction of planar areas based on the aggregation of spatially ordered lines [27] have also been developed, but the issue of outliers in segmentation has been re-highlighted, suggesting the need to improve the process of eliminating such data. These problems of depth data segmentation are addressed in our study, where we proposed a two-step filtering for the needs of reliable detection of outliers, which supports integration with the structure of online algorithms.

**Figure 2:**
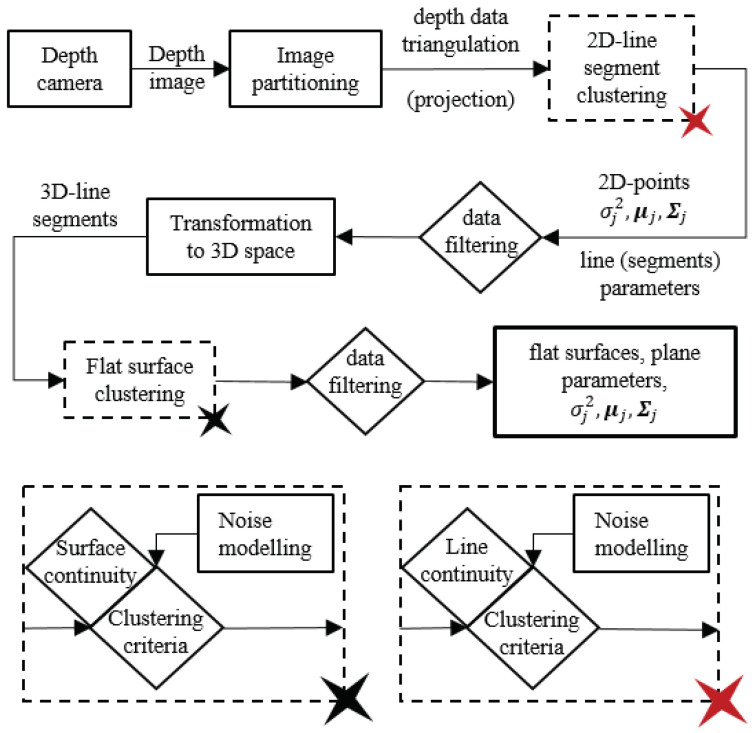
An overview of the EPCC-based segmentation.

In this paper, we present an extension of the EPCC [28] method to 3D space for the detection of flat surfaces in ordered depth maps (point cloud) that enables the parallelization of data-stream processing due to its structure. This study deals with the development of a perception algorithm for autonomous mobile systems based on the use of a depth camera. The perception algorithm is developed for indoor environments under controlled lighting conditions, which represents the limitations of the algorithm from the very beginning. Changing lighting conditions belong to the group of problems we encounter in the development of many perception algorithms. New sensor technologies, such as event cameras [29], have been developed to deal with challenging weather conditions. In this manner, we will present the importance of considering and modeling only part of the nonlinearity or uncertainty of the measurements [30] in order to provide more accurate results. Segmentation is performed on data from a depth camera using the recursive EPCC method, which involves the clustering of line segments and flat surfaces with linear prototypes, for which the main emphasis is on the recursive estimation of their parameters. Since depth map data are ordered, we can use data orderliness with the proposed approach to process a set of data in a single pass and to introduce a two-step filtering framework. This makes the algorithm suitable for real-time applications. Moreover, the presented method allows the consideration of the noise model as well as the non-homogeneous distribution of data in order to maintain the continuity of the segments. Figure 2 shows an overview of the proposed depth data clustering.

This paper is organized as follows. Section 2 gives a brief overview of the related work. In Section 3 and Section 4 are described the properties of the input data that dictate the structure of the segmentation algorithm. Section 5 describes the background of the line clustering, and noise modeling. In Section 6, the planar segmentation algorithm is described. Section 7 presents the results of the database-based experiments, and Section 8 presents the conclusions with discussion of the results.

## 2. Related Work

Grilli et al. [14] presented the methodologies used in point cloud segmentation. In our paper, we focused on the literature related to our work and highlighted three commonly used methods for plane detection in a point cloud: approaches based on RANSAC, region growing (RG), and the Hough transform. In addition, methods dealing with direct processing of depth images will be presented in Section 2.1.

### 2.1. Depth Image Segmentation Approaches

Methods for range image segmentation can be mostly categorized as region-based segmentation or edge-based segmentation methods [31]. Jiang and Bunke [32] presented a novel technique for determining planar patches in range images by grouping line scans, whereby the algorithm, based on the estimated fixed noise variance, can be adapted to different noise conditions. Lee et al. [33] proposed an image segmentation technique using an adaptive least k-th order squares (ALKS) estimator to minimize statistics of the squared of residuals. They highlighted a better tolerance to structured outliers. Koster and Spann [34] presented a statistical method to compare polynomial models with additive Gaussian distributed noise. They proposed a region-growing technique which uses the robust test to compare and merge adjacent regions. In addition to the planar model, the algorithm can be applied to segment 3D voxel data or to higher order vectorial data. Wang and Suter [35] presented model-based image segmentation algorithm based on hierarchical implementation and an Adaptive Scale Sample Consensus (ASSC) estimator. They highlighted the robustness of the algorithm to noisy or occluded data due to adaptive nature of the ASCC estimator. Muller and Brink [36] presented a method for segmenting range images into planar regions with graph cut optimization. The algorithm performs graph cuts in order to find an optimal configuration of normal vectors at all pixels. Hasnat et al. [37] proposed a novel unsupervised RGB-D segmentation method, which includes a joint clustering method on RGB-D features (color, position and normals) to generate a set of regions and a statistical planar region merging method to obtain final segmentation. Kumar and Ramakrishnan [38] presented a planar segmentation approach, which is based on Markov Random Field assumptions on depth data and solved using Graph Cuts. In this paper, we present a simplified, reliable and an evolving approach for the detection of planar regions in the case of noisy data, which is based only on exploiting the properties of an ordered data and an online calculation of its basic statistical properties.

### 2.2. RANSAC Approaches

The RANSAC (Random Sample Consensus) method was presented by Fischler and Bolles in [39] for the estimation of model parameters from experimental data that contain many outliers. Xu et al. [40] presented planar segmentation, in which they classified cloud points into different planar surfaces with the learned SVM (Support Vector Machine) models and SVM predictions. They compared their results with ordinary and NDT-RANSAC (Normal Distribution Transformation RANSAC) methods. Poz and Ywata [41] presented an adaptive approach to the roof segmentation of buildings, which includes both pre-processing of the point cloud for the separation of points between the associated buildings, as well as plane segmentation using the RANSAC method and the process of aggregation of over-segmented areas. Nguyen et al. [25] presented the segmentation of partially ordered LRF point clouds, in which they first segment scan profiles with a laser gauge based on directional vectors and then group these profiles to detect planes according to planar values of different neighborhood scan profiles.

### 2.3. Hough Transform Approaches

The Hough transform first introduced in [42] is, in addition to the RANSAC, a standard method for detecting parametric models. Vera et al. [43] presented the detection of planes in depth images for real-time operation using an implicit quadtree structure to identify clusters of approximately co-planar points in 2.5-D space. Khanh et al. [44] presented an improvement in ground segmentation in the point cloud for mobile robots based on the RHT (Randomized Hough Transform) combined with distance and angle constraints and compared it with the RANSAC method. Tian et al. [45] introduced a new method for segmenting planar properties in disordered point clouds based on extracted lines using a 2D Hough transform and an octree structure.

### 2.4. Region-Growing Approaches

The advantage of the RG method is that it takes into account properties of ordered point clouds and the neighboring point structure. Dong et al. [46] introduced a new hybrid method of the region growing, in which they presented the problem of point cloud segmentation as a robust optimization of global energy. The convergence of the algorithm or energy function was ensured by the simulated annealing approach. Wu et al. [47] presented the planar segmentation of Laser Range Finder point clouds using the MSTVM (Multiscale Tensor Voting Method) to better determine the point that represents the seed of the algorithm. They introduced a new property (the so-called plane strength indicator) to determine the seed point more intuitively. Huang et al. [48] introduced the EVBS algorithm (Encoding Voxel-Based Segmentation), which is based on the RG method by examining the structure of voxels and taking into account the limitations of continuity and smoothness.

## 3. Linear Prototype-Based Segmentation

In this paper, we focus on 3D planar segmentation of the point cloud (depth image), namely the extraction of flat surfaces based on the aggregation of detected line segments. The process is based on the EPCC method, which represents an evolving approach, in which we recursively estimate the parameters of linear prototypes that describe clusters of similar data. Linear prototype-based segmentation refers to the evolving clustering of data streams, in which clusters are described by its statistical properties and by the corresponding linear prototype. In the case of line segment detection, a line model, along with its statistical properties, is used to validate clusters expansion. The same approach is used in the detection of flat surfaces, except that the line model is replaced by a plane model.

### Integrating Data Structure in Evolving Clustering Framework

**Figure 3:**
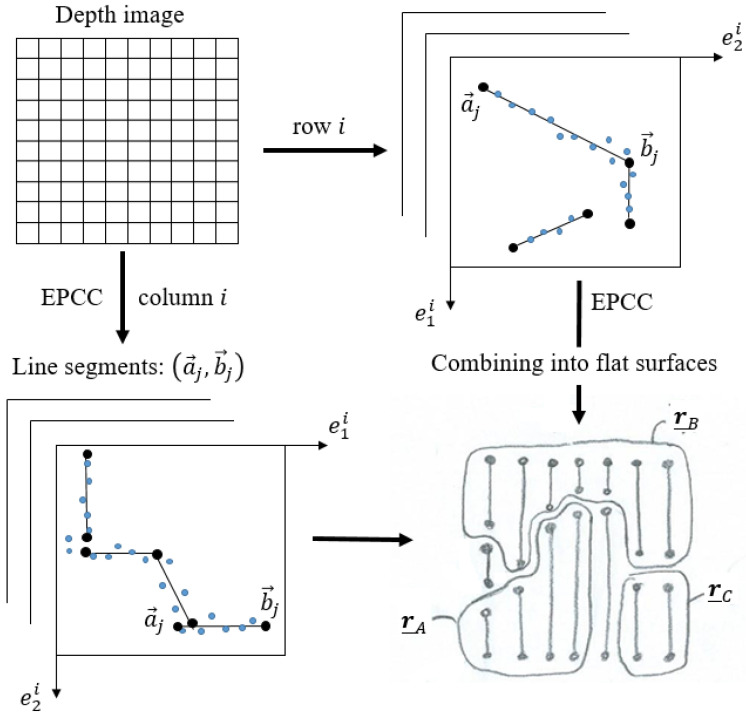
The concept of planar depth image segmentation with the EPCC method, in which the detection of 3D line segments takes place in 2D projections of columns or rows of the data matrix. Variables $e_{1}^{i}$ and $e_{2}^{i}$ represent the 2D coordinates of the points in the *i*-th column. With the process of line segment aggregation, we achieve a 3D-planar representation of the environment, where $\left\{{\underline{r}}_{A},{\underline{r}}_{B},{\underline{r}}_{C}\right\}$ represent flat surfaces or bounded planes determined by the start and end points $\left\{a_{j},b_{j}\right\}$ of line segments.

The method proposed in [28] is upgraded to work in structured indoor environments and enables integration with sensors, such as LRF and cameras, thus increasing the applicability of the method. In our case, we performed the experiments using an Intel RealSense D435i depth camera. In addition to recursiveness, the EPCC method is capable of exploiting data orderliness, so it is suitable for processing the data stream from the depth camera used, as its data structure allows independent processing of columns or rows of the data matrix, which also opens the door to process parallelization. The advantage of the used sensor and EPCC is that they allow one passage through the data or matrix columns that represent independent data sets. Additionally, the independent relations enable the use of multiple linear prototypes in the clustering framework, within which we can enable the integration of multiple-step filtering Figure 3 shows an overview of the depth image segmentation process, which takes place in columns (vertically) or in rows (horizontally) of the data matrix, independently.

The advantage of using such a sensor is in the structure of the output data, which is organized and enables the simplification of complex algorithms and even the parallelization of processes, on the basis of which we can enable real-time operation. The data structure itself allows for planar segmentation based on the use of two types of high-level features (lines and planes), which allows for an improved process of detecting outliers within a two-step filtering framework (Figure 2). Additionally, the use of linear prototypes has proven to be an excellent choice in the reconstruction of less textured surfaces, especially since we do not take into account additional information such as color. Taking into account the properties of the ordered data in the framework of the EPCC method enables online implementation, as the EPCC has an evolving character that allows real-time adaptation to data of different variances.

## 4. Image Partitioning Based on Input Data Properties

**Figure 4:**
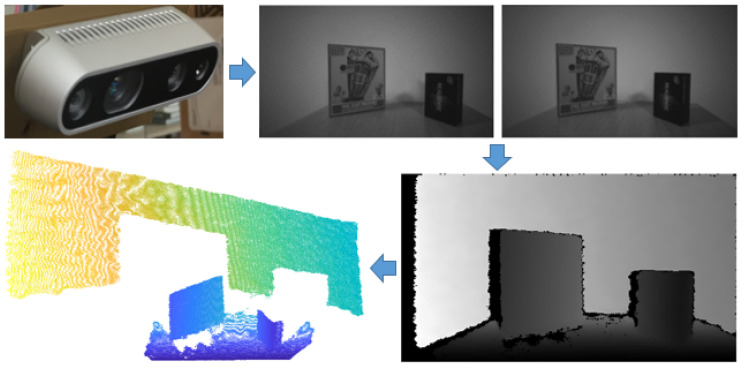
Acquiring of a depth image through inverse disparity, determined by corresponding point pairs of the left and right images.

The proposed method for line segment clustering is based on the use of input data properties obtained with the depth camera. The stereo camera sensor, using a specific method of finding the corresponding pairs of points in the left and right images, computes a disparity image, which can be converted into a depth image with the relation

(1) $$z=\alpha fbd^{-1}$$

where *z* is the depth value, α is the scaling factor, *f* is the focal length in pixels, *b* is the stereo depth baseline, and *d* is the disparity. The sensor output therefore represents a depth image or matrix of ordered depth points $Z=[z_{rc}],\;r=1,\ldots ,m,\;c=1,\ldots ,n$, whose dimensions represent the resolution of the image m×n and for which each element $z_{rc}$ represents a distance by Equation (1). The depth image can be converted into a suitable 2D/3D representation of spatial points (Figure 4), by considering the coordinate system of the camera, where the *z*-axis points out of the camera, the *x*-axis is in the horizontal direction and the *y*-axis is in the vertical direction. This is achieved by triangulation of depth data by Equation (2), taking into account the information about the image optical centre $\left(c_{x},c_{y}\right)$ in pixels. 3D points are stored in the matrix $H\in R^{m}\times R^{n}\times R^{3}$. The definition of the matrix *H* will be used later for comparison, which will confirm the hypothesis about the orderliness of the data obtained with the depth camera.

(2) $$z=z_{rc}\;x=\frac{(c-c_{x})\;z}{f}\;y=\frac{(r-c_{y})\;z}{f}$$

**Figure 5:**
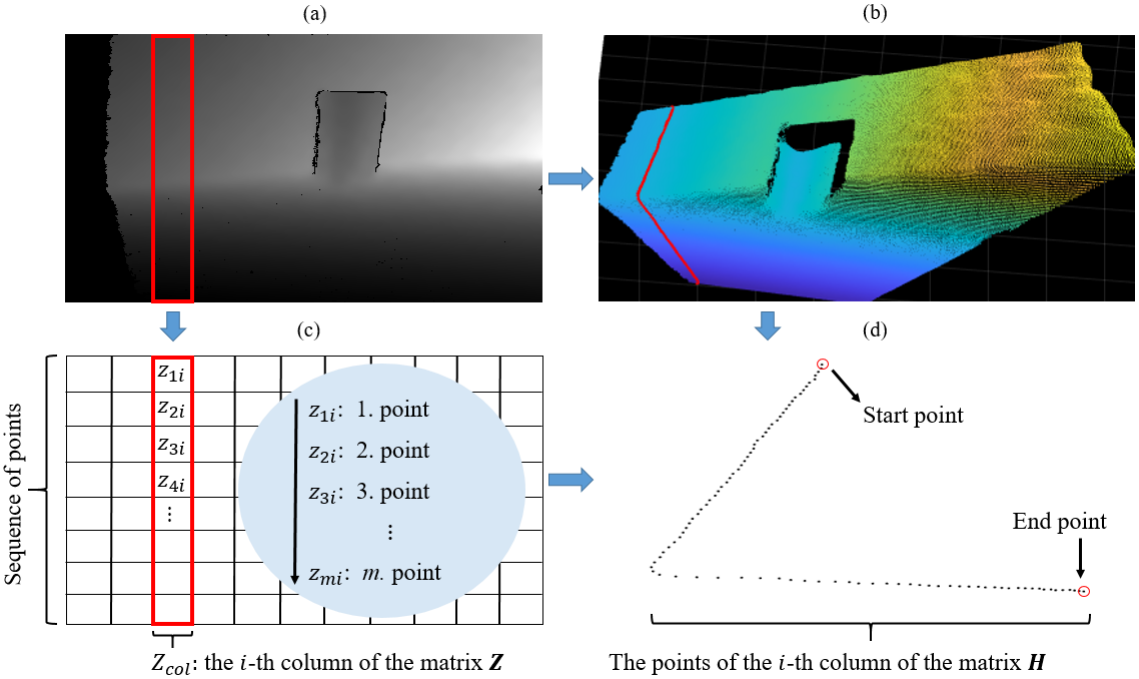
Depth image (**a**) and the corresponding point cloud (**b**). (**c**) shows the matrix form of the depth image, where the points in each column are arranged in order from the first to the last row. Display of 3D points of the highlighted column of the data matrix (**d**), which confirms the hypothesis about the orderliness and sequential occurrence of the data.

Due to the way the data are acquired, the points in the matrix *Z* (consequently in *H*) are locally ordered. In addition to the orderliness, we also observe a pattern of successive occurrence of points within individual columns and rows of the data matrix (Figure 5). Furthermore, we can observe that points (within columns), which are colored red in Figure 5, on a plane represent the planar section of the canonical field of view of the depth camera. The orderliness and sequence of points are important properties that give our algorithm a structural and methodological form.

A by-product of the given properties is the independent treatment of columns or rows $Z_{col}\in R^{m}\times R^{1}$ of the *Z*. This means that the algorithm can be structurally simplified and accelerated; simultaneously, the possibility of process parallelization opens up. The latter is very important as we want to keep the EPCC functioning in real time. In the continuation of the paper, only the segmentation by columns $Z_{col}$ of the data matrix *Z* is discussed, since by transposing it we can also achieve column segmentation of horizontal data, which is enabled by the structure of the proposed algorithm. We use the independence of matrix columns to partition *Z* in the case of line detection, and thus translate the problem of batch clustering to the treatment of individual partitions $Z_{col}$ independently of others. Separation between partitions is not a problem for the algorithm, as the dimensions of the input matrix are known in advance.

### 4.1. Line Segment Search Space

**Figure 6:**
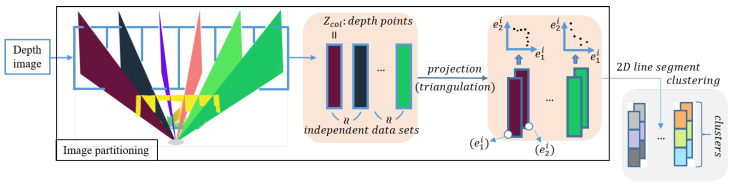
Depth image points practically lie on color coded planes sections, which means that the lines can be detected in 2D projections.

The data structure, or the nature of capturing spatial points within the cameras (conical) FOV, enables the detection of line segments with line prototypes in 2D projections of the point cloud. As shown in Figure 5 and Figure 6, the points of the matrices $Z_{col}$ lie on the plane sections of the camera’s FOV that can serve as a search space for line segments.

The individual $Z_{col}$ or $H_{col}\in R^{m}\times R^{1}\times R^{3}$ thus represents a plane on which 3D points can be projected while maintaining the geometric relations between them. The latter can be solved by introducing a new local coordinate system of that plane, in which each individual 3D point must follow the equation

(3) $$h_{col}\left(k\right)=h_{0}+t_{1}\cdot e_{1}+t_{2}\cdot e_{2}$$

where *k* is the index of the current point in $H_{col}$, $t_{1}$ and $t_{2}$ are 2D coordinates of the point $h_{col}\left(k\right)$ in the plane’s coordinate system with $h_{0}$ being its origin. The coordinate axes of the plane section are determined by orthonormal directions $e_{1}=\left(e_{x1},e_{y1},e_{z1}\right)$ and $e_{2}=\left(e_{x2},e_{y2},e_{z2}\right)$, which must meet the following conditions

(4) $$n\cdot e_{1}=0\;n\cdot e_{2}=0\;e_{1}\cdot e_{2}=0$$

where *n* is the plane’s normal (plane section). This way of projecting data can be a time-consuming process, which can also have difficulty determining orthonormal directions in the case of sparse data. We can do this in a more refined and faster way, which is described in Section 4.1.1.

#### 4.1.1. Plane Section as a 2D Search Space

**Figure 7:**
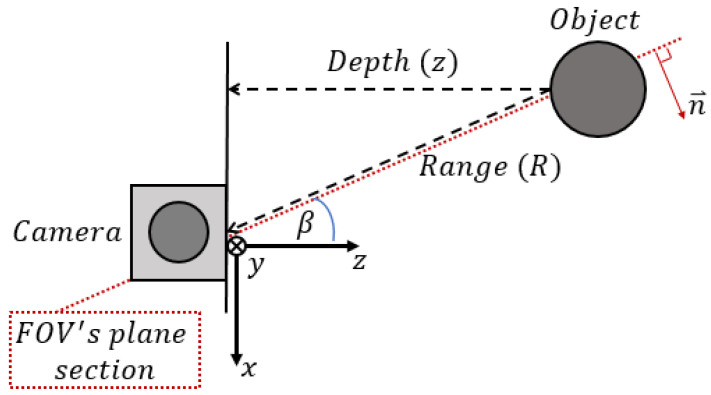
Depth vs range. Every depth pixel value is a measurement from the parallel plane of the imaging devices (i.e., a pair of cameras) and not the absolute range to the object.

Since the camera model and dimensions of the image are known, we can simplify and accelerate the process of determining 2D points by performing projection (i.e., triangulation) on the fly directly from the depth image *Z* by reintroducing Equation (2)

(5) $$\begin{array}{cc} z= & z_{rc}\;x=\tan\left(\varphi \right)z_{rc}\;y=\tan\left(\psi \right)z_{rc}\\ & \tan\left(\varphi \right)=\frac{c-c_{x}}{f}\;\tan\left(\psi \right)=\frac{r-c_{y}}{f} \end{array}$$

and by considering the depth-range relation defined by Equation (6), as shown in Figure 7.

(6) z=Rcos(β)

where β can be determined from φ and ψ

(7) $$\tan^{2}\left(\beta \right)=\tan^{2}\left(\varphi \right)+\tan^{2}\left(\psi \right)$$

**Figure 8:**
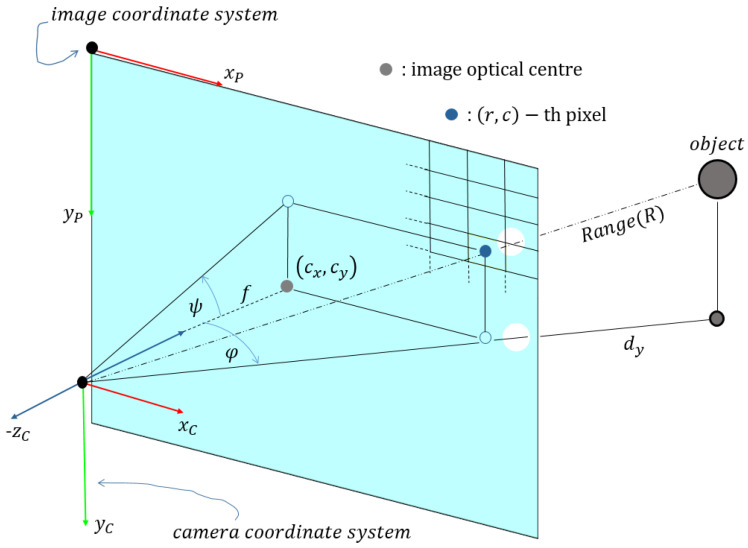
The triangulation technique as a tool for straightforward 2D point projection or simulation of the synthetic data.

The updated aspect of triangulation equations is shown in Figure 8, where 2D-coordinates are defined as

(8) $$\left(t_{1},t_{2}\right)=\left(d_{y},y_{c}\right)$$

where $d_{y}$ is the distance from the $y_{c}$-axis and can be obtained by considering horizontal offset φ of the spatial point (Figure 8)

(9) $$d_{y}=\frac{z}{\cos(\varphi )}=z\sqrt{1-\tan^{2}\left(\varphi \right)}$$

**Figure 9:**
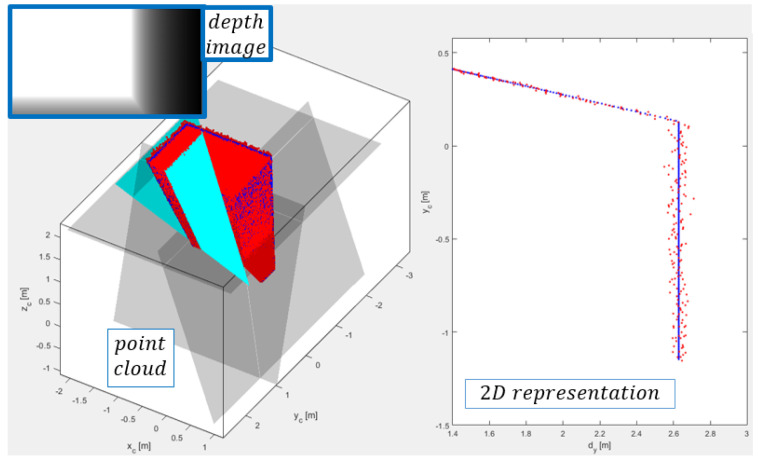
Simulated depth image and the corresponding point cloud on the **left** side. On the **right** side is demonstrated an example of the 2D representation of spatial points, on a plane section, which is represented as a set of planar rays. Ideal data are colored blue and corrupted data red.

With synthetic data, we can evaluate the performance of custom algorithms on ideal or corrupted data with arbitrary noise or, if necessary, tweak their structural form. Figure 9 shows a synthetically generated depth image (point cloud)

(10) $$z\left(r,c\right)=\frac{-d}{a\;\tan(\varphi (r,c))+b\;\tan(\psi (r,c))+c}$$

where (a,b,c,d) are user-defined plane parameters.

## 5. Evolving Line Segment Clustering

For the sake of transparency, we will introduce a new variable $T\in R^{m}\times R^{n}\times R^{2}$ for the input data matrix, whose columns $T_{col}\in R^{m}\times R^{1}\times R^{2}$ represent the 2D equivalent of 3D points of the matrices $H_{col}$. Once the 2D representation of the depth image is defined, we can start segmenting the image by clustering the points $t_{j}\left(k\right)$, within each partition $T_{col}$, into line segments. Segmentation in each 2D projection is performed using recursive estimation of the statistical properties of 2D data

(11) $$\sigma_{j}^{2}\left(k_{j}\right)=\frac{k_{j}-2}{k_{j}-1}\sigma_{j}^{2}\left(k_{j}-1\right)+\frac{1}{k_{j}}d_{j}^{2}\left(k\right)$$

(12) $$\mu_{j}\left(k_{j}\right)=\frac{k_{j}-1}{k_{j}}\mu_{j}\left(k_{j}-1\right)+\frac{1}{k_{j}}t\left(k_{j}\right)$$

(13) $$\begin{array}{cc} \; & \Sigma_{j}\left(k_{j}\right)=\frac{k_{j}-2}{k_{j}-1}\Sigma_{j}\left(k_{j}-1\right)\\ \; & +\frac{1}{k_{j}}\left(t\left(k_{j}\right)-\mu_{j}\left(k_{j}-1\right)\right){\left(t\left(k_{j}\right)-\mu_{j}\left(k_{j}-1\right)\right)}^{T} \end{array}$$

where $\sigma_{j}^{2}$ is the variance of the orthogonal distance $d_{j}\left(k\right)$ to the *j*-th linear prototype, $\mu_{j}\in R^{2}$ is the average value of the *j*-th cluster, $t(k_{j})$ is the 2D-point belonging to *j*-th cluster and $\Sigma_{j}\in R^{2\times 2}$ is the covariance data matrix of the *j*-th cluster. A linear prototype is described by line parameters that the proposed method evaluates directly from the covariance matrix $\Sigma_{j}$ using only basic arithmetic operations, which allows acceleration of the algorithm. The *j*-th model of the linear prototype is therefore described by the normal vector or eigenvector $p_{j}$, which belongs to the smallest eigenvalue of the matrix $\Sigma_{j}$

(14) $$\lambda_{j}=p_{j}^{T}\Sigma_{j}p_{j}$$

**Figure 10:**
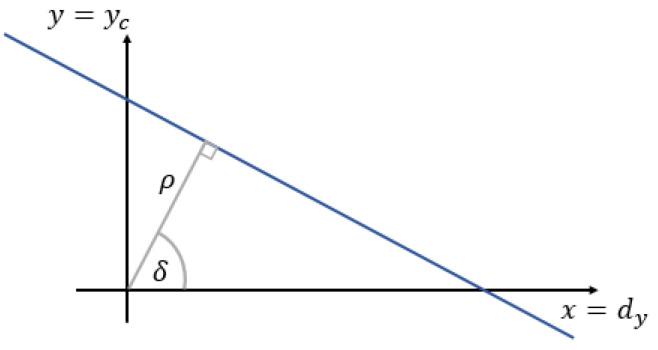
The normal form of the equation of a line.

Equation (15) describes the normal vector, which is obtained from the matrix $\Sigma_{j}$

(15) $$p_{j}=\left\{\begin{array}{ccc} {\left[\frac{\theta }{\sqrt{1+\theta^{2}}},\frac{-1}{\sqrt{1+\theta^{2}}}\right]}^{T}\; & ; & |\lambda_{1}\left|\leq \right|\lambda_{2}|\\ {\left[\frac{1}{\sqrt{1+\theta^{2}}},\frac{\theta }{\sqrt{1+\theta^{2}}}\right]}^{T}\; & ; & |\lambda_{1}\left|>\right|\lambda_{2}| \end{array}\right.\;$$

where θ and eigenvalues $\lambda_{1}$ in $\lambda_{2}$ are determined by

$$\theta =\frac{-\sigma_{11}^{2}+\sigma_{22}^{2}+\sqrt{\sigma_{11}^{4}+\sigma_{22}^{4}-2\sigma_{11}^{2}\sigma_{22}^{2}+4\sigma_{12}^{4}}}{2\sigma_{12}^{2}}$$

$$\lambda_{1}=\sigma_{22}^{2}-\theta \sigma_{12}^{2}$$

$$\lambda_{2}=\sigma_{22}^{2}-\theta \sigma_{12}^{2}+\frac{1+\theta^{2}}{\theta }\sigma_{12}^{2}$$

and where $\sigma_{io}^{2},i,o\in \left\{1,2\right\}$ are the elements of the covariance matrix. To avoid singularity cases, the line parameters can be estimated using parameters δ and ρ, which can describe an arbitrary line written in normal form (see also Figure 10)

(16) xcos(δ)+ysin(δ)=ρ

Normal vector is therefore defined as

(17) $$p_{j}^{T}=\left[\begin{array}{cc} \;\cos(\delta ) & \;\sin(\delta ) \end{array}\right]$$

where δ and ρ can be computed with frequently used orthogonal least-squares approach [49]

(18) $$\left[\begin{array}{c} \rho \\ \delta \end{array}\right]=\left[\begin{array}{c} x\;\cos(\delta )+y\;\sin(\delta )\\ \frac{1}{2}\arctan\frac{-2\sigma_{12}^{2}}{\sigma_{22}^{2}-\sigma_{11}^{2}} \end{array}\right]$$

where $\sigma_{12}^{2},\sigma_{22}^{2},\sigma_{11}^{2}$ are elements of Equation (13).

The algorithm clusters the data within $T_{col}$ by initializing a new cluster, placing it on the active list, and expanding it according to certain criteria, which are described below. Cluster spreading is halted when the data do not meet the criteria and are removed from the active list. In this way, we always expand those clusters that were last discovered, which is also made possible by information about the orderliness and successive appearance of the data. The criterion for adding to the active or *j*-th cluster depends on the orthogonal distance to the *j*-th line model

(19) $$d_{j}\left(k\right)=\left|{\left(t\left(k\right)-\mu_{j}\right)}^{T}p_{j}\right|$$

where t(k) is the current data sample and $\mu_{j}$ is the mean value of the *j*-th cluster’s data.

**Figure 11:**
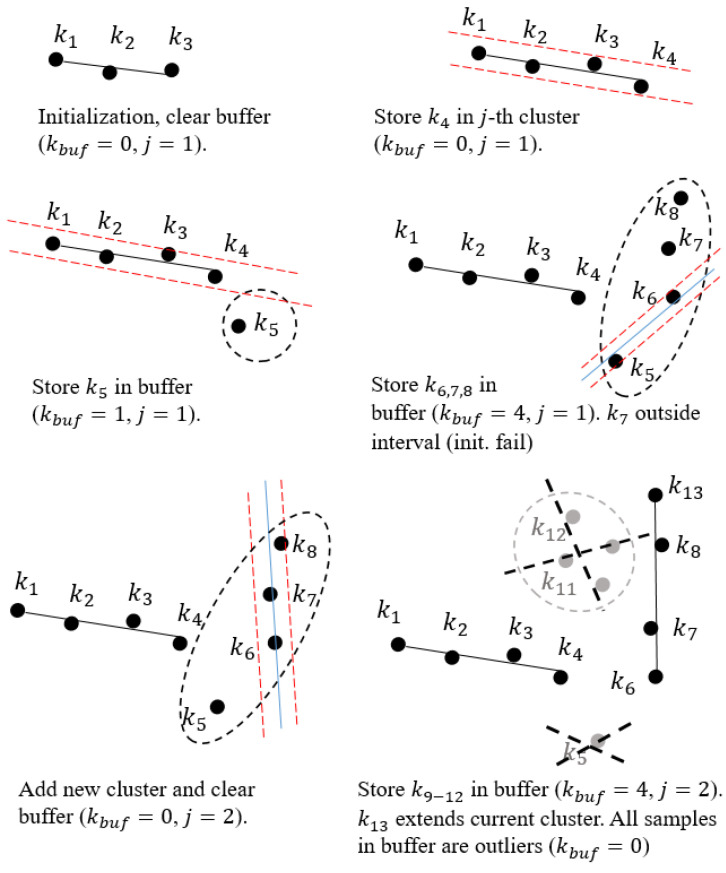
Illustration of the line segment clustering operation. The first $k_{min}=3$ samples initialize the first cluster. The next sample extends the first cluster if it is sufficiently close to the straight line. Samples $\left(k_{5}:k_{8}\right)$ fall outside the acceptance interval and therefore are buffered until $k_{min}$ collinear and consistent samples form a new cluster. Sample $k_{5}$ is characterized as an outlier when a new cluster is found. Next, samples ($k_{9}:k_{12}$) are buffered due to the failed extension of the active cluster and by the confirmed active cluster extension with sample $k_{13}$.

Noise is present in the acquired data, which increases with the square of the distance. To enable robust data clustering, the presence of noise needs to be considered. For this purpose, we introduce into the clustering criteria the definition of the variance of the orthogonal distance of all points in the *j*-th cluster $\sigma_{j}$ and the noise model $\sigma_{z}$, which depends on the depth

(20) $$d_{j}\left(k\right)<k_{max}\sqrt{\sigma_{j}^{2}+\sigma_{z}^{2}\left(z\right)}$$

where $k_{max}$ represents positive constant, which determines the sensitivity of the clustering criteria in the case of normal noise distribution ($k_{max}=3$ would imply in proper classification of 99.7% of all samples). Each cluster expansion, with new data sample, is followed by an update of the normal vector and the statistical characteristics of the clusters. Figure 11 illustrates a brief overview of line segment clustering as presented in [28].

### 5.1. Line Segment Extraction Algorithm

The line segment detection pseudocode is presented in the Algorithm 1.

**Figure 12:**
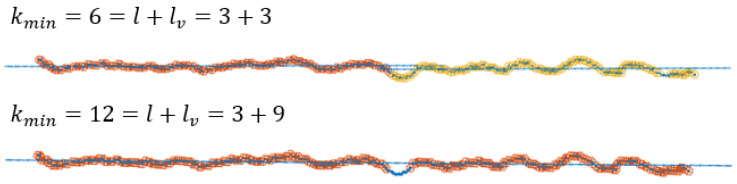
The effect of increasing $k_{min}$ and $n_{buf}$ is shown in the greater probability of outlier detection and in the reduction in over-segmentation according to Equation (20), where $k_{max}=2$.

The output of the algorithm is highly dependent on the choice of input parameters, as they determine the shape of the searched segments. Furthermore, the setting of the optimal parameters depends on the sensor used. In this section, an approximate estimate of the range of values for the input parameters, in the case of using a depth camera, is given. In the case of using LRF, the parameter settings are given in [28]. The parameter $k_{min}=l+l_{v}$ specifies the minimum number of points needed to reliably build a new cluster prototype, with *l* being the number of points used to build the model and $l_{v}$ the number of points that must meet the clustering criteria. As introduced in [28], parameter $k_{min}$ determines dimensionality *l* of the input data, with which a linear model can be estimated from at least *l* data samples. Following the example of [28], for the purpose of robust segmentation, in the case of noise and outliers, it is necessary to set a higher parameter $k_{min}$, so that the identification of linear models becomes over determined. The appropriate selection of the $k_{min}$ (including $n_{buf}$) plays an important role in segmentation over longer distances (Figure 12), as the noise increases with the square of the distance. The minimum line segment size is a matter of application, but at longer distances it is recommended to choose a larger $k_{min}$, thus detecting larger line segments, as this is the only way that the correct segments can be successfully detected. By changing the ratio between *l* and $l_{v}$, we can improve the treatment of outliers as well as the proportion of noise in the data in the initialization process, where $l_{v}>l$. The values for *l* and $l_{v}$ must meet the following requirements $\left\{l\geq 2,\;l_{v}\geq 0\right\}$. By selecting $l_{v}=0$, we disable the data consistency check in initialization step. The extreme increase in $k_{min}$ can have negative consequences, i.e., under-segmentation.
**Algorithm 1:** Line segment detection with the EPCC method.1: Parameter definition: $k_{min}$, $k_{max}$, $n_{buf}$ = 2$k_{min}$, initialize buffer and list of prototypes 2: **for** k=1:m 3:    Calculate $d_{j}\left(k\right)$ of the sample t(k). 4:    **if** $d_{j}\left(k\right)\leq k_{max}\sqrt{\sigma_{j}^{2}+\sigma_{z}^{2}}$**then** 5:        Add t(k) to the *j*-th cluster, update Equations (11), (12), (13) and (15).            Delete previous data in buffer buf. 6:    **else** 7:       Store t(k) in buf. 8:       **if** $length\left(buf\right)\geq k_{min}$**then** 9:             Find new a cluster candidate from buf and estimate $d_{j}$ of the $l_{v}$ next points from buf. 10:            **if** $n_{good}\geq l_{v}$ next points in buf are consistent 11:                Initialize the prototype, set j=j+1 and update $\mu_{j}$, $\Sigma_{j}$, $p_{j}$ and $\sigma_{j}$.                     Clear buffer. 12:            **else** 13:                 if $len\left(buf\right)\geq n_{buf}$, remove the oldest sample 14:            **end if** 15:      **end if** 16:   **end if** 17: **end for** 18: Outputs→ordered list of line segments and their properties 


The $n_{buf}$ parameter is used to set the maximum number of points in the buffer buf that are still being considered in the cluster detection process. The role of the buffer is, in addition to the clustering criteria, to remove potential outliers. This is done by simply removing the last data sample in buffer when the number of points in it exceeds $n_{buf}$. Outlier removal can be enabled only when $n_{buf}$ is higher than $k_{min}$. The setting of the buffer parameters, in the case of dealing with laser data, is comprehensively described in [28]. By changing the input parameters, we can vary the size of the smallest detected line segment. Instructions for selecting proper $k_{min}$ and $n_{buf}$ are presented in Table 1.

**Figure 13:**
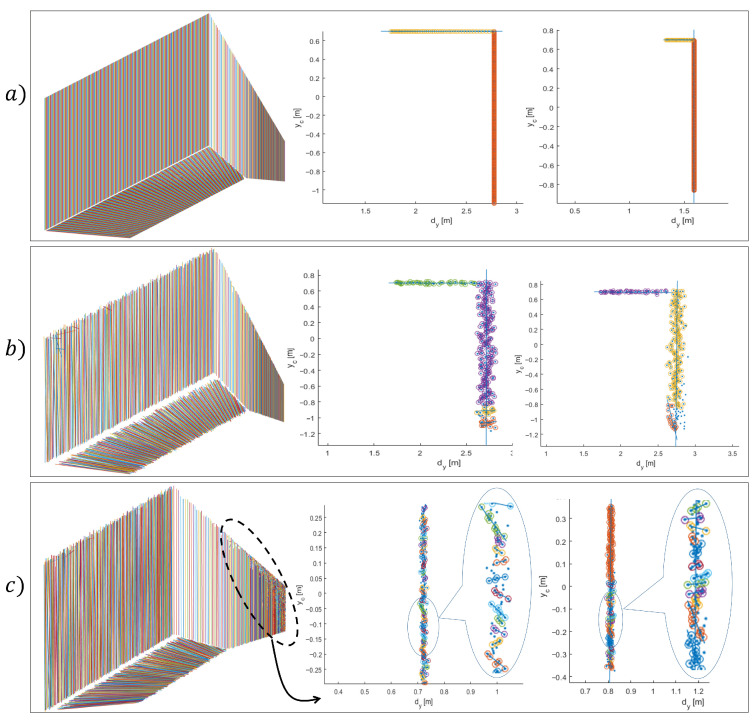
Line segment detection on ideal data (**a**), which also confirms the proposed methodological and structural form of data processing; (**b**) shows an example of detection on corrupted data, which produces low over-segmentation but still succeeds in detecting representative line segments with appropriately selected input parameters that are given in Table 1; (**c**) example of the high rate of over-segmentation when $k_{min}$ is small ($l=2,l_{v}=1$).

**Figure 14:**
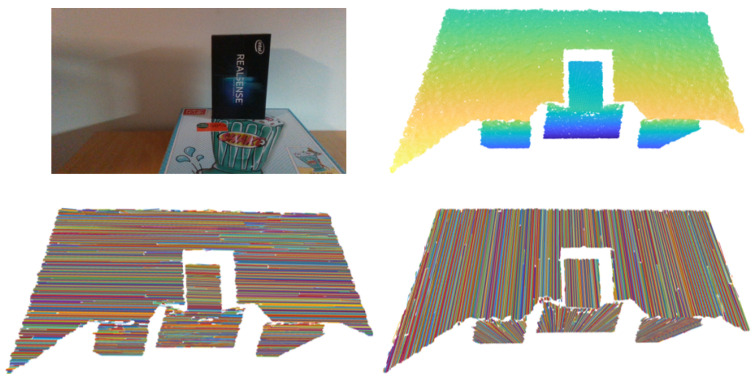
RGB scene and point cloud (**upper** pictures). An example of line clustering within columns of the depth image, i.e., vertical clustering (**bottom right** picture). The same applies for rows, since by transposing the depth image we achieve horizontal clustering (**bottom left** picture).

The algorithm’s outputs are identified 2D line segments that are defined by the linear prototype’s parameters (eigenvectors and clusters centers), statistical properties, and endpoints. Since we are dealing with a sorted data stream, these points represent the first $a_{j}$ and last point $b_{j}$ of an individual cluster. To retain the efficient evolving approach, only endpoints of line segments can be stored in the whole process of clustering. Additionally, these points can be projected onto the corresponding lines with respect to their orthogonal distances $d_{j}$ to the linear prototype

(21) $$a_{j_{p}}=a_{j}-d_{j}p_{j}\;b_{j_{p}}=b_{j}-d_{j}p_{j}$$

where $a_{j_{p}}$ and $b_{j_{p}}$ stand for the *j*-th projected endpoints. Projection consequently changes the original depth map or *z*-coordinate (distance) and needs to be recomputed when transforming points to 3D space with Equation (9), then *x*-coordinate with Equation (5) and finally $y=y_{c}$. Figure 13 demonstrates the algorithm’s performance on ideal synthetic data and data corrupted with noise, similar to one described in the section “Noise Modelling”. An example of vertical and horizontal line clustering is demonstrated in Figure 14.

### 5.2. Noise Modelling

Data exposure to noise can lead to poor segmentation results in the form of over-segmentation and a loss of representative segments. This problem can be mitigated by taking noise into account. Noise in stereo sensors usually reflects quadratic behavior and has been often described as a function of measured distance [50,51,52]. Thus, we introduce the noise model into the clustering criteria defined by Equation (20)

(22) $$\sigma_{z}\left(z\right)=k_{1}z^{2}+k_{2}z+k_{3}$$

where $k_{1}=0.006954$, $k_{2}=-0.003713$ and $k_{3}=0.001153$. The noise model is evaluated after dynamic sensor calibration using Intel’s dynamic calibration tool.

By observing the axial distribution of noise, Sung et al. [30] showed that it can be modelled with the Gaussian distribution. Axial noise is considered to be the pixel-wise standard deviation of the orthogonal distances between the triangulated points and an estimated vertical plane, provided by the white target. The experiment is performed by placing the white target at known distances and performing an analysis of the aforementioned covered distances.

**Figure 15:**
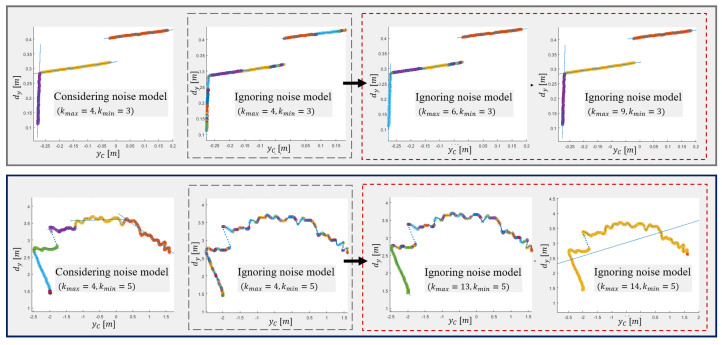
Line segment detection on real data acquired with D435i. Two examples showing how taking noise into account reduces over-segmentation and the possibility of incorrect clustering by reducing an increase in $k_{max}$. In dashed rectangles the results when disregarding modelled noise during segmentation are shown. Based on the results in the red rectangles, we can conclude that in the case of using a depth camera, we can ignore quadratic noise behavior at smaller distances, where over-segmentation is reduced by increasing $k_{max}$, but it can fail at larger ones due to its significant increase.

The examples in Figure 15 show that taking noise into account further improves segmentation performance. In addition to reducing over-segmentation, with Equation (20) taking noise into account, we can reduce the probability of incorrect clustering. The latter can occur when we want to optimize $k_{max}$ and increase its share of acceptable data by increasing it. By considering Equation (22), we reduce the need for an unwanted increase in $k_{max}$, which can also lead to the segmentation of genuine segments. The presented model cannot compensate the depth point estimation error that occurs when searching for a disparity image.

## 6. Flat Surface Detection

The flat surface detection algorithm retains the methodological form of the line segment detection algorithm, in which the orthogonal distance of the current data sample to the linear prototype was used to determine its membership to one of the already existing clusters but slightly differs in implementation. When processing, Algorithm 1 maintains the data’s ordered and sequential appearance, which is reflected in the ordered occurrence of 3D line segments, which, in addition to their statistical properties, represent the input of a flat surface detection algorithm. In our case, this data arrangement dictates the algorithm’s structure, which is set to recursively estimate 3D linear prototypes. To keep the idea of 3D segmentation simple, we only use endpoints of line segments. In this section, a method of investigating ordered data (line segments) is presented, which has the capability to establish the second filtering step and the real-time operation by single-pass learning. Additionally, an analysis of the algorithm parameter setting is performed for determining the optimal parameters. Moreover, in the name of intuitive parameter setting, an analysis on how the modelled data properties influence the desired outcome and help reduce the tuning effort, is introduced.

**Figure 16:**
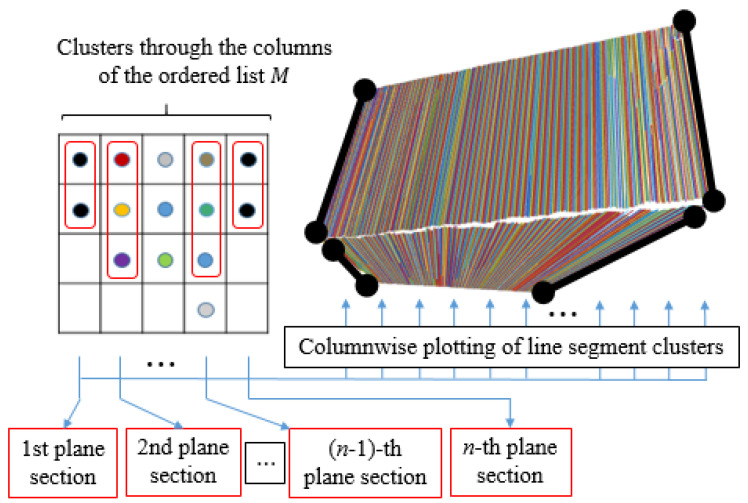
List *M* of ordered line segments and their visualization. So called columns of the list represent line segments lying on individual planar sections and are determined by their endpoints.

**Figure 17:**
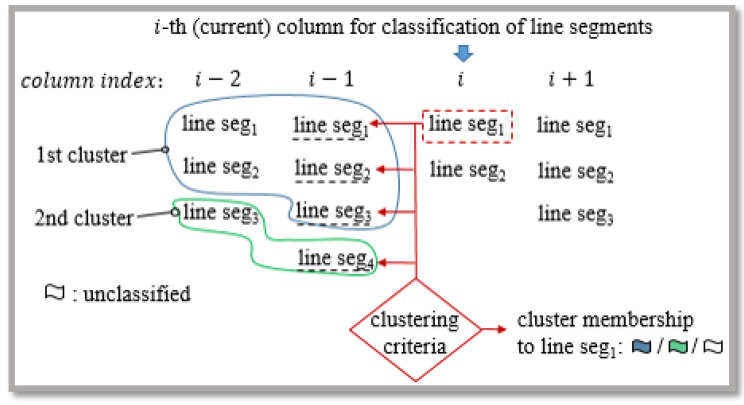
Flat surface clustering using line segments.

Flat surface detection contains two steps: cluster initialization and its propagation through the ordered list *M*, where each element represents a 3D line segment and its statistical properties (Figure 16). The operation of the algorithm can be described as an aggregation of line segments between adjacent planar sections of the camera’s FOV or between the columns of list *M* as shown in Figure 16 and Figure 17.

In an algorithmic sense, the cluster propagation through adjacent columns of M is carried out according to the current column index (Figure 17). A line segment of the current column is classified to the *j*-th cluster, present in the previous column, if the orthogonal distance $\left(d_{j1}\left(k\right),d_{j2}\left(k\right)\right)$ of the endpoints to the *j*-th linear prototype meets criteria

(23) $$d_{j}\left(k\right)<k_{max}\sqrt{\sigma_{j}^{2}+\sigma_{z}^{2}\left(z\right)+\sigma_{seg}^{2}\left(k\right)}$$

where $\sigma_{seg}^{2}\left(k\right)$ is the current line segment’s residual, i.e., the distance variance obtained from Algorithm 1, $\sigma_{j}^{2}$ is the distance variance of the *j*-th propagated cluster and $d_{j}\left(k\right)$ is the orthogonal distance to the prototype

(24) $$d_{j}\left(k\right)=\left|{\left(h-\mu_{j}\right)}^{T}p_{j}\right|$$

where *h* is a 3D-Cartesian endpoint of a line segment, $\mu_{j}\in R^{3}$ is the average value of the *j*-th cluster and $p_{j}\in R^{3}$ is the prototype’s normal vector. The segmentation process is again based on a recursive assessment of statistical properties defined by Equation (11), (12) and (25), this time in the case of 3D data, for which normal vector $p_{j}$ is calculated using singular value decomposition of the covariance matrix $\Sigma_{j}\in R^{3\times 3}$. To robustify the clustering process, the recursive estimation of covariance matrix is weighted and is defined as

(25) $$\begin{array}{cc} \; & \Sigma_{j}\left(k_{j}\right)=\frac{k_{j}-2}{k_{j}-1}\Sigma_{j}\left(k_{j}-1\right)\\ \; & +\frac{w}{k_{j}}\left(t\left(k_{j}\right)-\mu_{j}\left(k_{j}-1\right)\right){\left(t\left(k_{j}\right)-\mu_{j}\left(k_{j}-1\right)\right)}^{T} \end{array}$$

where

(26) $$w=e^{\frac{-d_{j}{\left(k\right)}^{2}}{\mu_{j_{d}}^{2}}}$$

and where $\mu_{j_{d}}$ is the average orthogonal distance to the *j*-th linear prototype.

The correct classification of the current line segment into a true cluster is achieved by checking all combinations, as shown in Figure 17. This is done by using the current line segment (from the column indexed with *i*) to find the best criterion (Equation (23)) with respect to all line segments in the previous column (indexed with i−1) and to determine the membership to the appropriate cluster. This has proved to be necessary, especially in maintaining the continuity of flat surfaces. There are many holes in the point cloud (depth image) that can represent dividers between surfaces. We maintain the continuity of these by introducing criteria with Equation (27) that take into account the minimum Euclidean distance $d_{evk_{min}}$ of the current line segment to the related cluster

(27) $$d_{evk_{min}}+\sigma_{z}\left(z\right)\leq d_{1}$$

where $d_{1}$ is constant, which determines the upper limit for $d_{evk_{min}}$. In this way, we can prevent the classification into a cluster, where there is an adequacy to the Equation (23) but the assumption of surface continuity is violated. Each (*j*-th) propagated cluster is tracked by introducing the membership labels or IDs, which are illustrated with different contour colors in Figure 17. Thus, we can verify the identity of the clustered line segments in the adjacent (i−1)-th list column.

**Figure 18:**
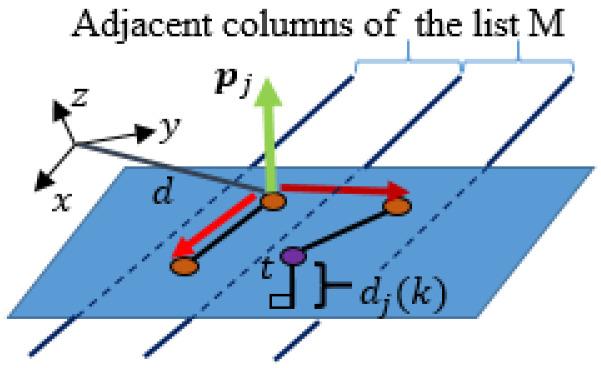
Initialization of a prototype plane with points of two unclassified line segments of adjacent columns of the list *M* with respect to the orthogonal distance to the plane.

The initialization of new prototypes is carried out when we attempt to discover a new cluster or the expansion of existing ones is not possible. Initialization is the formation of a new linear prototype $p_{j}$ with three endpoints of line segments located between adjacent columns of the list M (Figure 18).

As shown in Figure 18, the prototype is then validated according to Equations (23) and (27) where $d_{j}\left(k\right)$ is the orthogonal distance of the fourth point to the linear prototype. To reliably form a new cluster, the $k_{max}$ can be lowered (e.g., by 50%) and we can check if the directional vectors of the line segments have approximately the same orientation, i.e., the angle between them is less than a certain threshold.

During clustering, some line segments may remain unclassified. Those that remain unclassified in the *i*-th column go to the new cluster initialization process in the next step. However, those that remain unclassified in the i−1-th column become outliers. Algorithm 2 presents the plane detection pseudocode. Because there is a small number of elements (Algorithm 2), the proposed search is not very time consuming.
**Algorithm 2:** Detection of planes by the EPCC method.1: Parameter definition: $d_{1}$, $k_{max}$. 2: **for** i=1:c (number of *M*’s columns) 3:     **for** $k=1:r_{1}$ (num. of rows of the *i*-th (current) column) 4:         **for** $s=1:r_{2}$ (num. of rows of the (i−1)-th column)                  Cluster current $seg_{i}\left(k\right)$ to the *j*-th cluster with the best Equation (23) of all combinations 5:              **if** $\left\{d_{j1}\left(k\right),d_{j2}\left(k\right)\right\}\leq k_{max}\sqrt{\sigma_{j}^{2}+\sigma_{z}^{2}+\sigma_{seg}^{2}\left(k\right)}\;\;\&\;\;d_{evk_{min}}\leq d_{1}\;$**then** 6:                  Add $seg_{i}\left(k\right)$ to the *j*-th cluster, update Equation (11), (12) and (25), $p_{j}$ and exit the loop 7:              **end** 8:              **if** classification failed **then**
                    (find a new cluster among the unclassified segments) 9:                  **if** $d_{j}\left(k\right)\leq k_{max}\sqrt{\sigma_{z}^{2}+\sigma_{seg}^{2}\left(k\right)}\;\;\&\;\;d_{evk_{min}}+\sigma_{z}\left(z\right)\leq d_{1}$ 10:                     Add the new cluster and its label $\left(j=ID_{new}\right)$ and estimate                        $\sigma_{j}^{2}=d_{j}^{2}\left(k\right)$, Equations (12) and (25), $p_{j}$. 11:                 **end if** 12:             **end if** 13: 3× **end for**


**Figure 19:**
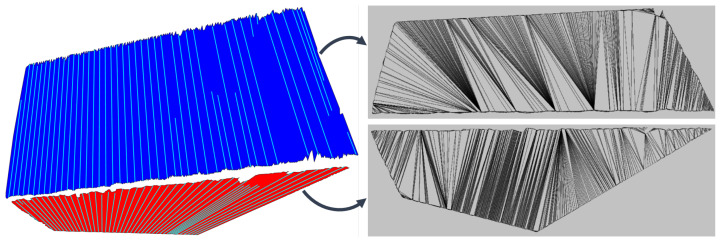
Bounded flat surfaces of a point cloud (**left**), whose boundaries are determined by projected endpoints of line segments. Clusters are shown where every tenth corresponding line segment is drawn. Triangle meshes of surfaces are shown on the (**right**).

The output of the proposed algorithm is clusters of line segments that represent 3D flat surfaces, their statistical properties, and corresponding parameters of linear prototypes. These surfaces can be of any shape, as their shape is determined by the start and end points of the line segments. The detected clusters of planar points $c_{j}$ have a certain deviation from the corresponding linear prototypes. These deviations represent the orthogonal distances $d_{j}$ of points to the prototypes $p_{j}$, which are taken into account when projecting onto a plane

(28) $$c_{j_{p}}=c_{j}-d_{j}p_{j}$$

where $c_{j_{p}}$ represents the *j*-th cluster of projected planar points (line segments). Figure 19 shows the result of Algorithm 2, where the planar points are projected onto the corresponding planes.

It is important to note that the structure of the planar segmentation algorithm is tuned to exploit the ordered arrangement of line segments, which is also preserved in individual clusters after the clustering process. With this information, we can more quickly and easily determine the contour of the surface or the edges of the object (Figure 19), determined from the endpoints of the line segments. An example of the use of contour points is shown in Figure 19, where the generation of a high-level geometric structure of concave shape is demonstrated using the sweep-line algorithm for constrained Delaunay triangulation [53].

### 6.1. Algorithm Tuning Effort

**Figure 20:**
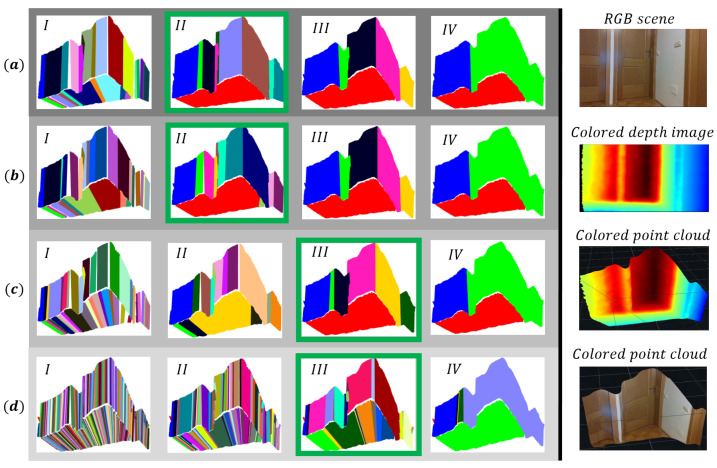
Clustering results for different forms of clustering criteria (**a**–**d**) and different values of $k_{max}$ for each scene, as presented in Table 2. The best segmentation results are marked with a green rectangle, where $k_{max}$ is set optimally. In this case, we still successfully detect flat surfaces but avoid incorrect clustering.

Algorithm 2 has two tuning parameters ($k_{max}$ and $d_{1}$), of which the $k_{max}$ has the greatest impact on clustering quality. Because the clustering criteria consider data variance, the clustering adapts to current data. This makes it robust to data scaling or to different noise in the data [28]. As shown in the operation of Algorithm 1, this reduces tuning effort and possibility of wrong clustering. In this section, we will show the impact of parameter tuning on clustering results as well as the results of using different forms of clustering criteria. Figure 20 shows the clustering results when tuning the parameter $k_{max}$, whose values are presented in Table 2.

The operation of the algorithm was tested for different forms of clustering criteria, for which we found the optimal values of $k_{max}$. With the proposed clustering criteria (Equation (23)), we achieve the lowest rate of over-segmentation with little tuning effort.

## 7. Experiments

The experiments were performed on a database obtained with the Intel RealSense D435i depth sensor, which represents structured interior scenes. Each scene is represented by a depth image of size 270×480; thus, a point cloud contains 129,600 spatial points. The proposed method is compared with the RG and RANSAC method, which are part of the Point Cloud Library [54]. In Table 3, the method’s parameters, which were set experimentally to obtain optimal results, are displayed and described. Our research is focused on solving problems of environmental perception with depth cameras for the purpose of autonomous navigation of mobile systems in large environments. The results will be evaluated with performance indicators, which indicate the over-segmentation and under-segmentation of areas and their correct detection. Therefore, we wish to make a comparison with the aforementioned methods, on the basis of which we want to justify the use of an evolving framework instead of standard working principles and demonstrate the cons and pros of each method. For the purpose of developing and validating the SLAM application in the future, a comparison on a public database will also be welcome. With the proposed method, we achieve piecewise planar patch representation of arbitrary objects.

### 7.1. Experimental Comparison

**Figure 21:**
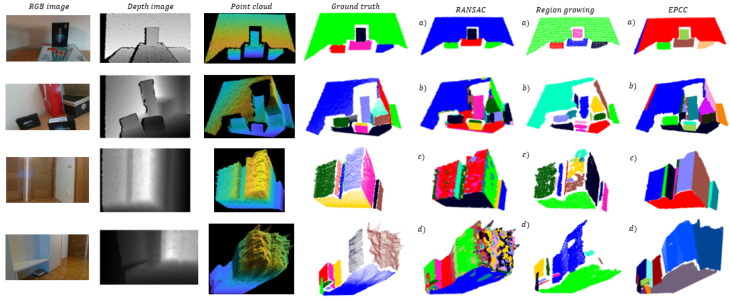
Planar segmentation results, in which individual flat surfaces of a point cloud are color-coded. Clustered region-growing point clouds are more sparse due to required filtering process. More importantly, we can observe that the EPCC gives comparable or better results over long distances (bottom two rows), where depth measurement errors are considerably high due to noise quadratic behavior.

Clustering results are evaluated in terms of the proportion of correctly detected flat surfaces $\frac{D}{N}$, where *D* is the number of correctly detected areas and *N* is the number of all flat surfaces in a given scene, which represent ground truth as illustrated in Figure 21. We say that a certain algorithm correctly detects a surface when it maintains its continuity, despite the possible over-segmentation or under-segmentation of the same. The number of identified clusters for each method is denoted as IC. We also estimate the proportion of over-segmented $\frac{N_{n}}{N}$ and under-segmented $\frac{N_{p}}{N}$ areas, where $N_{n}$ is the number of over-segmented and $N_{p}$ is the number of under-segmented areas. A particular area is not over-segmented when it is not fragmented or broken into several parts. If the algorithm successfully detects a flat surface, it is considered under-segmented if less than 80% of the points are classified according to ground truth.

In Figure 21, the results of planar segmentation of the methods are presented. In Table 4, the segmentation results for all the methods are shown. It is important to note that in the case of segmentation with the RG method, it was necessary to filter the data; otherwise, the method gives incomparable results, which are reflected in excessive over-segmentation. Using the VoxelGrid filter [54], we down-sampled a point cloud by approximating points inside each voxel with their centroid. It is important to emphasize that the tested RG and EPCC method have a set value of the minimum (maximum) cluster size. This means that after the segmentation is done, all clusters that have fewer points than minimum (or have more than maximum) will be discarded, which leads to a reduction in over-segmentation.

When comparing methods, it is first necessary to point out the essential difference between them, which will prove to be key in achieving good segmentation results. Compared to our method, the biggest disadvantage of the RG and RANSAC method is non-adaptability or inflexibility of the clustering criteria when processing point clouds. These methods have fixed parameters that need to be determined in advance for the needs of a particular situation or application. Similarly, when using the EPCC method, the input parameters of the algorithm must also be determined in advance (e.g., $k_{max}$ and $d_{1}$), but for further operation (in changing noise conditions) this is no longer necessary, as the clustering criteria are adaptive and have the ability to adapt to data variability. It follows that not much effort is required in setting the parameters, as mentioned in Section 6.1. Presetting the parameters in the EPCC can be done quite easily in most cases. Nevertheless, we must be careful when we are performing segmentation at shorter distances (scenes (a) and (b)), where (due to very low noise amplitudes) it is necessary to increase the input parameter $k_{max}$ (Table 3) to avoid over-segmentation. The adaptive character of the clustering criteria together with the consideration of the noise model plays an important role in the detection of flat surfaces over longer distances, where large noise amplitudes are often present in the case of stereoscopic environmental perception.

On the left Figure 21 shows a database representing (non) complex scenes whose objects are close as well as far from the depth camera origin. In the case of RG and RANSAC, we can see in Table 3 that it is necessary to reset a collection of parameters to achieve optimal results for each scene; RANSAC requires a smaller number of input parameters. The inflexibility of their criteria (parameters) as well as the disregard for the noise model leads to poorer segmentation results (Table 4), which can be quickly observed in Figure 21, in the case of detecting more distant objects in scene (d). This shows the sensitivity of the RG and RANSAC methods to noise; the former did not detect a door at the end of the corridor, and the latter described this part of the space with excessive data over-segmentation. Furthermore, a disadvantage of the RANSAC method is that it does not maintain the continuity of the surfaces, which can lead to overlapping of certain areas if complete areas have not been detected in the previous steps. In contrast, in the case of the EPCC method, we can observe from the results the desired segmentation effect over long distances (see Table 4), in which the EPCC achieves a high rate of successfully detected flat surfaces and identifies the fewest clusters. In addition, it achieves minimal over-segmentation and under-segmentation of flat surfaces.

The success of point cloud segmentation is also conditioned by the successful detection and exclusion of many outliers. RANSAC has been designed to work well in the case of data that contain many outliers, but due to the great uncertainty and nonlinearity of the measurements it fails to deliver complete reconstruction, as shown in scene (c) and (d). The same goes for the RG method, which appears to be even more sensitive to noise at given input parameters according to visually demonstrated results in Figure 21. This includes the inability to detect the surfaces of objects, or objects in their entirety, which are often accompanied by a high over-segmentation rate. In contrast, the outlier detection and its exclusion in our case is ensured through a two-step filtering, which is carried out recursively within the high-level feature detection, e.g., lines and planes. As demonstrated in the proposed 3D sensing method and in the other applications (e.g., SLAM), the use of high-level features again proved to be beneficial as they allow for error minimization and for assuring optimal results. The use of features in the evolving framework of the EPCC algorithm has proved promising, because in most cases it has enabled reliable detection of textureless homogeneous surfaces present in a given database. This is very important since passive stereo systems have difficulty in finding a disparity image when there is very little texture on the surfaces of the observed environment (e.g., a completely clean homogeneous plate or board). Active systems improve the accuracy of the measured depth by artificially creating texture on the scene by using structured light of its own origin. Although in most cases this considerably resolves the correspondence problem of stereo perception, these stereo setups are not entirely immune to visual ambiguities.

At this point, we need to highlight another advantage of the EPCC method. With the latter, we obtain not only the parameters of flat surfaces (polygons) recursively, but also the uncertainty of the parameters. Information about the uncertainty can play an important role in robotic applications, such as localization. In addition, the surfaces in our case can be represented as a set of ordered line segments (with the associated parameter uncertainties) or as a set of points, which can be either projected from the data or we can randomly (ordered) sample the bounded surface. In this way we can efficiently generate high-level geometric structures, such as meshes and dense surfaces, for the purpose of building various AR/VR applications.

The iterative nature of the algorithms (e.g., RANSAC, Hough-Transform) limits the applicability of certain methods to unorganized point clouds, while the EPCC algorithm is capable of processing ordered as well as general data streams, as stated in [28]. The advantage of the proposed method is its evolving nature, which is characterized by the ability to adapt to the variance of current data and different noise conditions. Theoretical and simulation explanation showed that the EPCC algorithm is capable of integration with other sensor systems that have a similar principle of data acquisition, which increases the applicability of the method. Based on the experimental comparison, we can observe that the EPCC achieves performance comparable to established methods for planar segmentation of point clouds on a given database. It is worth nothing that the performance of the proposed method strongly depends on the modelled properties of the input data. Changing light conditions and different depth camera orientations could significantly affect the performance of the algorithm.

### 7.2. Robustness to Soft Data Transitions and Noise

**Figure 22:**
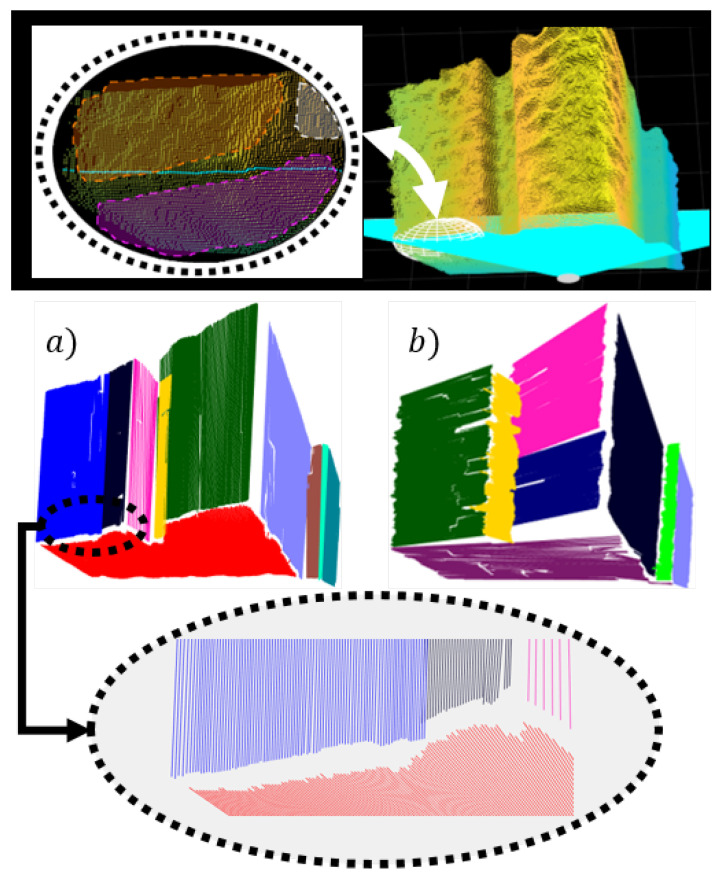
Horizontal scanning of a point cloud and an area of soft transitions between the boundaries of flat surfaces marked by a white ellipsoid (upper pictures). The intermediate area between colored flat surfaces represent data loss. The case of vertical clustering (**a**), for which the smallest data loss and highest accuracy is achieved. Horizontal clustering (**b**) is more affected by blurred boundaries, which caused under-segmentation of the violet colored floor and thus bigger holes.

Blurred boundaries between objects can reduce segmentation accuracy and cause the occurrence of holes between certain surfaces (Figure 22). This often happens due to soft or slow data transitions between the surfaces of different objects, which can make the segmentation of depth information difficult, as there are no sharp inter-object transitions due to large noise amplitudes, especially at greater distances. Locally unrecognizable boundaries between objects can also be the result of occlusions, including tough noise conditions. With certain scanning directions of the depth image, the borders may appear additionally obscured, which can lead to information loss in the line segment clustering phase (Figure 22). This problem can be mitigated to some extent by quasi-mimicking the different scanning directions of the image or point cloud, where noise model consideration also plays an important role.

**Figure 23:**
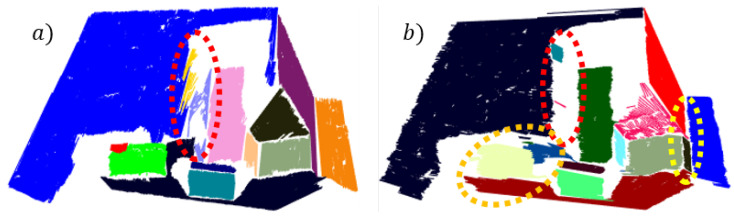
Red ellipse marks occluded sparse area which is over-segmented in vertical clustering (**a**) and almost completely removed in horizontal clustering (**b**). The floor is over-segmented due to occlusion caused by the object marked with orange ellipsis. Yellow ellipsis marks the part of the over-segmented red wall, as a result of blurred boundaries.

The advantage of the locally ordered data is that they enable arbitrary clustering direction, which can be utilized to solve the problem of transitions and noise. In our case, vertical and horizontal clustering represent two modes of the so-called depth image scanning. In Figure 22 the results for both cases are shown in the case of segmenting a distant scene with non-complex layout of the environment. In both cases, the clustered line segments are projected onto corresponding linear prototypes, which further increases hole size between adjacent surfaces. This problem can be addressed by triangulation between adjacent surfaces, where joint connections could be searched for by checking the possible intersections of adjacent line segments in the case of small holes. Moreover, the final boundaries of different surfaces can be also determined by considering additional information, such as texture or color. Figure 23 shows the comparison of horizontal and vertical clustering in the case of the nearby environment with complex layout and few occlusions.

**Figure 24:**
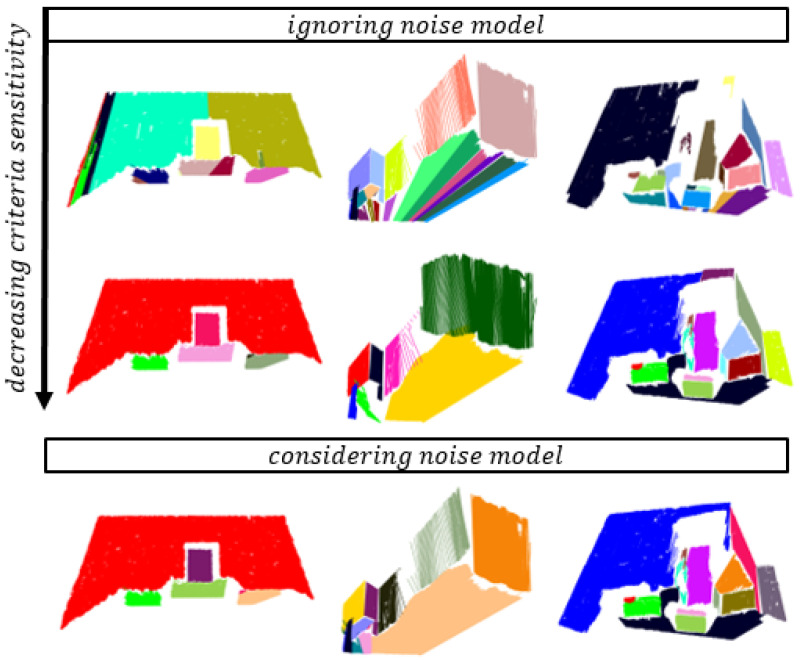
EPCC reduces over-segmentation by decreasing the sensitivity of clustering, which can inversely lead to increased sensitivity to the incorrect clustering, when disregarding the noise model in remote areas (**middle column**). This does not apply to nearby areas (**left** and **right**).

Figure 24 shows the robustness of the algorithm when taking noise into account. In low range areas, an increase in criteria parameters improves segmentation results despite disregarding the noise model, but fails in remote areas due to high noise amplitudes. This is usually a consequence of higher a probability of incorrect clustering, as demonstrated in Section 5.2.

## 8. Conclusions

We presented an evolving approach to point cloud segmentation based on the EPCC method, which recursively estimates statistical properties of clusters and corresponding linear prototypes’ parameters. The proposed method exploits data locality in an ordered data stream, which enables faster and simpler implementation and real-time data processing. The algorithm is upgraded to work in 3D space for the purpose of detecting flat connected surfaces based on an aggregation of line segments. The use of high-level features (e.g., lines and planes) enabled the detection of textureless homogeneous surfaces, which are known to cause visual ambiguities in stereo perception. Flat surfaces can be represented as a set of ordered line segments, which can simplify the extraction of a surface’s contour or edges of an object. In this manner, we can efficiently generate high-level geometric structures, such as meshes. We highlighted the possibility of arbitrary sampling of object surfaces, which can be used for generating dense representations. Moreover, the uncertainty of the linear prototypes’ parameters is obtained through recursive assessment of clusters’ statistical properties. This can play an important role in mobile robotic applications, such as localization.

The results showed that the EPCC approach can cope with established approaches for point cloud processing in terms of accuracy, even without prior data filtering. More importantly, it outperforms them over long distances when the signal-to-noise ratio is low due to the adaptive nature of clustering criteria. The RANSAC and RG methods require a lot of hard work in setting the parameters for each experiment in order to obtain optimal results. Moreover, the fixed nature of their clustering criteria is the main reason for poor performance in larger environments. We highlighted the problem of lighting conditions and the influence of camera orientation on data accuracy, which may limit the operation of the proposed method. On the given database, an average rate higher than 90% has been obtained for successfully detected flat surfaces. The robustness of the algorithm to data scaling and different noise conditions follows from the fact that the clustering criteria consider data variance, which allows the clustering to adapt to current data. The noise model, which is considered in the segmentation process, allowed for the compensation of characteristic uncertainties that are introduced into the measurements of depth sensors. In addition, noise consideration reduced the possibility of incorrect clustering and also reduced algorithm tuning effort. To ensure optimal results we introduced a two-step filtering for outlier detection within clustering framework.

We highlighted the problem of blurred boundaries between flat surfaces, which can lead to significant information loss. To alleviate this problem and the problem of occlusions, we proposed the mimicking of arbitrary scanning directions of the depth image, which was enabled by the fact that data appear locally ordered. The EPCC method is generally applicable as it allows the processing of ordered as well as general data streams where the data arrive randomly. Moreover, theoretical and simulation part proved that the algorithm allows for operation with other sensor systems that have similar data capture principles (e.g., laser range finders). Because the data structure allows separate searching by columns and rows of the depth image, we plan to explore the possibility of jointly combining data from columns and rows to describe planar features, which can form the basis for real-time SLAM or AR/VR application. In the future, we plan to include the proposed method in the SLAM application, the operation of which will be evaluated on a public data set.

## Figures and Tables

**Table 1:** Parameter selection instructions.

Parameter	Requirements	Recommendations
$k_{min}$	$\left\{l\geq 2,\;l_{v}\geq 0\right\}$	$4\leq k_{min}\geq 12$. Selection depends on data and desired segment size. In the case of noisy data from the depth camera it is recommended to choose the smallest possible value to reduce algorithm complexity, but one big enough to enable reliable detection of distinct surfaces on greater distances (e.g., $7\leq k_{min}\geq 10$).
$n_{buf}$	$n_{buf}>k_{min}$	if $n_{buf}=3k_{min}$ then correct cluster initialization can be obtained if the buffer contains less than ≈33% outliers

**Table 2:** Values of $k_{max}$ in the tuning process for different forms of clustering criteria. Bold numbers represent optimal values, where we achieve the lowest over-segmentation (see also Figure 20).

Example	Clustering Criteria			
(a)	$d_{j}\left(k\right)<k_{max}\sqrt{\sigma_{j}^{2}+\sigma_{z}^{2}\left(z\right)+\sigma_{seg}^{2}\left(k\right)}$			
$k_{max}$	1	**2**	3	4
(b)	$d_{j}\left(k\right)<k_{max}\sqrt{\sigma_{j}^{2}+\sigma_{z}^{2}\left(z\right)}$			
$k_{max}$	1	**2**	3	4
(c)	$d_{j}\left(k\right)<k_{max}\sqrt{\sigma_{j}^{2}+\sigma_{seg}^{2}\left(k\right)}$			
$k_{max}$	1	2	**3**	4
(d)	$d_{j}\left(k\right)<k_{max}\sqrt{\sigma_{j}^{2}}$			
$k_{max}$	1	2	**3**	4
Scene	*I*	II	III	IV

**Table 3:** Description of input parameters.

Param.	Value				Description	Method
*l*	1cm		3cm	4cm	leaf size (VoxelGrid filter)	RG
$c_{th}$	0.3		0.5	0.3	curvature threshold	
$\Theta_{th}$	$3^{\circ }$		$4^{\circ }$	$3^{\circ }$	smoothness constraint	
$num_{k}$	30				point normal estimation with *k* neighbors	
$min_{c}$	50				minimum cluster size	
$max_{c}$	$10^{6}$				maximum cluster size	
$k_{n}$	50				segmenting with *k* closest neighbors	
ine $d_{th}$	1.5cm		3.2cm	5cm	distance threshold
$w_{n}$	0.02			0.03	normal distance weight	RANSAC
$it_{max}$	100				max iterations	
$k_{min}$	8				see Section 5	EPCC
$k_{max}$	2(Algorithm 1)					
$min_{c}$	30					
$k_{max}$	3		2			
$d_{1}$	15cm					
Scene	(a)	(b)	(c)	(d)		

**Table 4:** Segmentation results.

Method	IC	*D* ($\frac{D}{N}$)	$\frac{N_{n}}{N}$	$\frac{N_{p}}{N}$	*N*
(a)					
RG	6	5 (100%)	0.0	0.2	5
RANSAC	11	5 (100%)	0.4	0.0	
EPCC	7	5 (100%)	0.4	0.0	
(b)					
RG	16	11 (100%)	0.09	0.18	11
RANSAC	32	9 (82%)	0.27	0.09	
EPCC	15	11 (100%)	0.09	0.0	
(c)					
RG	15	8 (89%)	0.22	0.33	9
RANSAC	25	9 (100%)	0.66	0.0	
EPCC	10	9 (100%)	0.1	0.0	
(d)					
RG	11	5 (56%)	0.0	0.22	9
RANSAC	53	8 (89%)	0.56	0.11	
EPCC	14	8 (89%)	0.22	0.0	
